# Remdesivir in COVID-19: pros and cons

**DOI:** 10.3389/fphar.2026.1731244

**Published:** 2026-02-12

**Authors:** Yara Rouhana El Feghali, Layan Rabih, Jad Abdul Khalek, Mariam Arabi

**Affiliations:** 1 Faculty of Medicine, American University of Beirut Medical Center, Beirut, Lebanon; 2 Division of Pediatric Cardiology, Department of Pediatric and Adolescent Medicine, American University of Beirut Medical Center, Beirut, Lebanon

**Keywords:** antiviral therapy, congenital heart disease, coronavirus, COVID-19, remdesivir, SARS-CoV-2

## Abstract

**Background:**

Beginning in late 2019, the COVID-19 pandemic caused by SARS-CoV-2 rapidly evolved into a global health crisis. High rates of severe illness, hospitalizations, and long-term complications highlighted an urgent need for effective therapeutic agents. This necessity drove unprecedented efforts in drug discovery and repurposing. Remdesivir, developed by Gilead Sciences in 2009, was initially designed as a broad-spectrum antiviral targeting Ebola virus disease. Following observations of broad antiviral activity against coronaviruses, remdesivir was granted Emergency Use Authorization by the FDA in May 2020 for hospitalized patients with severe COVID-19. The FDA subsequently issued full approval in October 2020, expanding remdesivir’s use to hospitalized adults and pediatric patients aged 12 years or older and weighing at least 40 kg.

**Aim:**

This paper aims to assess the advantages and limitations of remdesivir in the treatment of COVID-19, drawing on evidence from clinical trials and examining its application in patients with congenital heart disease (CHD).

**Methods:**

The literature review was conducted until September 2025 using PubMed and Google Scholar searching for recent clinical trials in addition to relevant reviews.

**Results and Conclusion:**

Remdesivir has been shown to shorten recovery time and lower mortality risk, particularly in patients at an early stage of infection with mild disease severity or requiring oxygen support. Although early guidelines advised against its use in patients with severe renal impairment, subsequent studies confirmed its safety prompting an FDA label update to allow use regardless of renal function. While some trials reported limited effects, the overall body of evidence supports remdesivir’s role in improving clinical outcomes in COVID-19 treatment. In patients with CHD, the uncertain effects of both COVID-19 and remdesivir highlight a key research gap, emphasizing the need to refine existing therapies while following National Institutes of Health (NIH) treatment guidelines.

## Introduction

COVID-19, a pandemic that claimed millions of lives and disrupted every aspect of society, stands as one of the greatest challenges of our time (Lippi et al., 2023). By the end of 2022, the disease had spread to every corner of the globe, with more than 650 million confirmed cases of SARS-CoV-2 infection and over 6.6 million COVID-19–related deaths (Lippi et al., 2023). What began in December 2019 as a cluster of acute atypical respiratory infections in China rapidly escalated to a worldwide crisis (Pagani et al., 2023). Initial cases were epidemiologically linked to an animal market, suggesting a zoonotic origin (Pagani et al., 2023). However, it soon became clear that SARS-CoV-2 was capable of efficient human-to-human transmission, primarily through respiratory droplets (Rahman et al., 2021; Bala et al., 2021).

SARS-CoV-2 is a novel coronavirus belonging to the family Coronaviridae (Fernandes et al., 2022). Genomic analysis revealed that the positive-sense single-stranded RNA virus was 96% homologous to Bat-CoV RaTG13 virus, supporting a zoonotic origin, and 80% similar to the SARS-CoV virus responsible for the 2002–2003 outbreak (Zhou et al., 2020).

Clinically, COVID-19 presents with a broad range of illnesses, from asymptomatic cases to severe respiratory failure requiring intensive care (Piccicacco et al., 2022; Sharma et al., 2021). Most commonly, patients present with typical symptoms including fever, dry cough, dyspnea, fatigue, myalgia, and loss of taste and smell (Ochani et al., 2021; Mohamadian et al., 2021). Several risk factors have been associated with an increased morbidity and mortality from COVID-19, including older age, male sex, and pre-existing comorbidities (Zhang et al., 2023). Among these comorbidities, cardiovascular disease, such as congenital heart disease (CHD), has emerged as an important determinant of COVID-19 severity due to underlying hemodynamic and cardiopulmonary vulnerability (Ehwerhemuepha et al., 2022). Although COVID-19 vaccines have demonstrated strong protective effects and have been widely incorporated into routine immunization programs, SARS-CoV-2 has continued to evolve with variants that exhibit increased transmissibility and partial immune escape, highlighting the persistent need for effective therapeutic interventions (Scott et al., 2025; Organization, 2022; Roederer et al., 2024). Breakthrough infections and severe disease continue to occur, particularly among older adults, immunocompromised individuals, and patients with multiple comorbidities (Lipsitch et al., 2022). Consequently, antiviral agents remain essential for reducing viral replication, limiting disease progression, and improving clinical outcomes in high-risk and hospitalized patients (Niknam et al., 2022).

Treatment strategies for COVID-19 have since evolved with the aim of controlling viral replication, alleviating hyperinflammation and providing supportive care. Standard supportive measures include hydration, oxygen supplementation, and mechanical ventilation in critical cases (Gavriatopoulou et al., 2021). Moreover, therapeutic strategies have emerged including antiviral drugs, immunotherapies, convalescent plasma, and nanoparticle-based therapeutics (Majumder and Minko, 2021). One of the most widely studied antiviral agents is remdesivir (Veklury), a broad-spectrum nucleotide analogue that targets the viral RNA-dependent RNA polymerase (RdRp), thereby inhibiting viral replication (Kokic et al., 2021). In May 2020, remdesivir was the first drug to be granted Emergency Use Authorization by the FDA for the treatment of hospitalized COVID-19 patients (U.S. Food and Drug Administration, 2020a). This was followed by full FDA approval in October 2020, extending its use to adults and pediatric patients (≥12 years of age, weighing at least 40 kg) requiring hospitalization (U.S. Food and Drug Administration, 2020a).

Although remdesivir was rapidly implemented, research has shown conflicting findings regarding its efficacy. While some clinical trials reinforced its effectiveness through its ability to reduce viral load by two folds and likelihood of hospitalization, others failed to show significant benefits in reducing hospital stay and mortality especially in vulnerable populations with advanced forms of the disease (Piccicacco et al., 2022; Pan et al., 2021; Lingas et al., 2022). Furthermore, some clinical trials reinforced the use of remdesivir in combination with other drugs. For instance, combining it with baricitinib has shown to improve clinical outcomes; whereas studies with tocilizumab did not demonstrate any significant improvement (Rosas et al., 2021; Kalil et al., 2021). Other clinical trials faced limitations such as small sample size and methodological constraints (Brown et al., 2023).

These gaps highlight the need for comprehensive evaluation to establish the role of remdesivir as an effective treatment for COVID-19. In particular, patients with CHD represent a vulnerable subgroup, as underlying structural cardiac abnormalities and altered cardiopulmonary physiology may predispose them to more severe infection and adverse outcomes (Haiduc et al., 2021). Emerging evidence suggests that individuals with CHD may experience higher rates of hospitalization, complications, and prolonged recovery following SARS-CoV-2 infection (Haiduc et al., 2021). In this context, antiviral therapies, including remdesivir, may have distinct safety and efficacy profiles in patients with CHD. This paper aims to assess the benefits and drawbacks of using remdesivir for COVID-19 treatment, based on evidence from clinical trials that have tested this therapeutic agent, while also considering its use in patients with CHD.

## Methods

The screening for randomized controlled trials was carried out until 25 June 2025 using the PubMed and Google Scholar databases. To find the relevant trials exploring the use of remdesivir in COVID-19 patients, the results were retrieved using the following keywords: (“COVID-19“ OR “SARS-CoV-2“) AND (“remdesivir“ OR “antivirals“).

The literature review was continued until September 2025. Pertinent review papers were incorporated to ensure a thorough examination of remdesivir (history of development and pharmacology) and SARS-CoV-2 (epidemiology, pathophysiology, clinical manifestations, and treatments). To gather information about the pharmacology of remdesivir, the keywords used were “remdesivir“ AND (“pharmacology“ OR “pharmacokinetics“ OR “pharmacodynamics“ OR “mechanism“). Furthermore, articles addressing COVID-19 in patients with CHD were included as a distinct focus of this paper. To reduce publication bias and ensure completeness, clinical trial registries were also screened (ClinicalTrials.gov and the WHO International Clinical Trials Registry Platform [ICTRP]) for remdesivir trials; registry records were used to identify potentially relevant studies and confirm trial characteristics. Trials without publicly available results were not included in outcome synthesis. Human studies published in English (original or translated), evaluating remdesivir in patients with COVID-19, were included. For efficacy assessment, priority was given to randomized controlled trials (RCTs), including pragmatic randomized studies. For safety evaluation and special populations (e.g., patients with renal impairment, pediatric populations), high-quality observational studies and secondary analyses were included when RCT data were limited. Eligible studies were required to report at least one clinical or safety outcome, such as time to recovery, ordinal scale improvement, hospitalization, need for supplemental oxygen or mechanical ventilation, mortality, or renal, hepatic, or cardiac adverse events.

In vitro-only and animal-only studies were excluded from the clinical efficacy synthesis, as were duplicate reports, editorials or commentaries without original data, and registry entries without publicly available results. Titles and abstracts were screened for relevance, followed by full-text review. Extracted data included study design, population characteristics, disease severity, remdesivir regimen and timing, comparators, and outcomes. Discrepant findings were reconciled using prespecified factors—timing of initiation, baseline severity, endpoint selection, and statistical power—to synthesize consistent patterns across disease stages rather than relying on individual trials.

## Pharmacology

**FIGURE 1:**
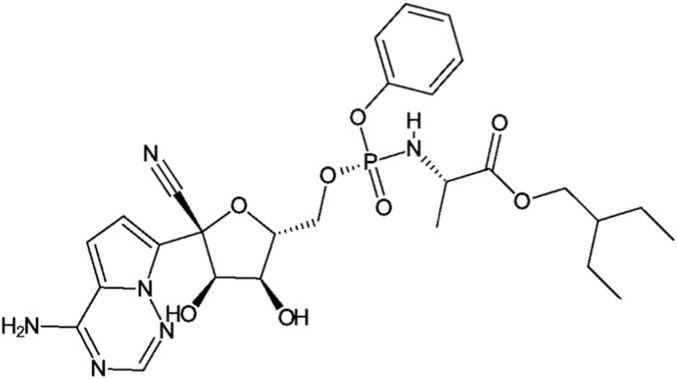
Chemical structure of remdesivir (GS-5734) generated in ChemDoodle from PubChem data (CID: 121304016).

Remdesivir, marketed under the brand name Veklury, has the empirical formula C_27_H_35_N_6_O_8_P, a molecular weight of 602.6 g/mol, and a CAS number of 1809249-37-3 (Royal Society of Chemistry, 2025). The two-dimensional structure of remdesivir is illustrated in Figure 1.

### Pharmacodynamics

As the prodrug of a 1′cyano-substituted adenosine nucleotide analogue, remdesivir (GS-5734) exerts its action within host cells (Nabati and Parsaee, 2022). It is mostly metabolized by carboxylesterase 1 or to a lesser degree by cathepsin A and CYP3A, yielding the nucleoside monophosphate intermediate GS-704277 (Humeniuk et al., 2021; Bakheit et al., 2023). Upon cleavage of the phosphoramidate bond in the intermediate, the nucleoside monophosphate GS-441524-MP is released and subsequently phosphorylated to generate the pharmacologically active nucleotide triphosphate metabolite GS-443902 (Bakheit et al., 2023). In parallel, GS-441524-MP is also dephosphorylated to its parent nucleoside analogue GS-441524 which becomes the major circulating metabolite (Bakheit et al., 2023; Wang et al., 2022).

As an adenosine triphosphate analogue, remdesivir inhibits viral RNA synthesis by targeting the SARS-CoV-2 RdRp (Blair, 2023). Although it is the major enzyme for viral transcription and replication, RdRp requires contributions from non-structural proteins (Malone et al., 2022). Nsp7, nsp8, and nsp12 form the RdRp holoenzyme with nsp12 enclosing the RdRp domain responsible for catalyzing RNA synthesis (Malone et al., 2022).

It was initially thought that the inhibition of RNA synthesis by remdesivir exclusively occurs via a “delayed chain termination“ mechanism due to the molecular structure of the drug (Deb et al., 2021). This means that after remdesivir’s insertion into the growing chain of viral RNA, a steric clash between the drug’s nitrile group and Ser-861 of RdRp hinders the enzyme’s translocation halting viral replication (Malone et al., 2022). However, *in vitro* studies have shown that this mode of inhibition can be overcome by nucleoside triphosphate concentrations below physiological levels suggesting the existence of another mechanism (Tchesnokov et al., 2020).

**FIGURE 2:**
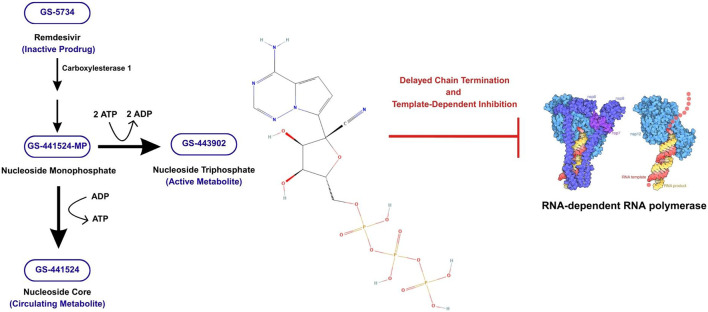
Remdesivir mode of action (created on Canva; RNA-dependent RNA polymerase (RdRp) provided by https://pdb101.rcsb.org/motm/249 and GS-443902 molecule provided by https://pubchem.ncbi.nlm.nih.gov/compound/56832906). This figure summarizes the intracellular activation and antiviral mechanism of remdesivir. Beginning on the left side of the figure, the inactive prodrug remdesivir GS-5734 undergoes sequential enzymatic activation. It is initially metabolized by carboxylesterase-1, after which further enzymatic processing yields the nucleoside monophospahte GS-441524-MP. This intermediate follows two pathways: (i) phosphorylation to form the active triphosphate GS-443902 (ii) dephosphoryation into the major circulating nucleoside metabolite GS-441524. Represented by the central chemical structure, the active triphosphate GS-443902 interacts with the viral RdRp. It disrupts viral RNA synthesis via delayed-chain termination and template-dependent inhibition as depicted on the right side of the figure.

Recent studies have shed light on “template-dependent inhibition,“ suggesting that remdesivir’s insertion into the template strand may impact the complementary RNA strand (Tchesnokov et al., 2020). Due to steric hindrance with Ala-558, remdesivir is misaligned with the template strand, which inhibits the addition of uridine triphosphate into the complement strand (Tchesnokov et al., 2020). Finally, remdesivir’s effectiveness stems from the RdRp enzyme’s more favorable selectivity for the drug over endogenous adenosine triphosphate, facilitating its incorporation into viral RNA strands (Blair, 2023). Figure 2 presents a schematic summary of the mode of action of remdesivir.

Remdesivir has exhibited broad-spectrum activity against multiple RNA virus families *in vitro* and in preclinical models (Santoro and Carafoli, 2021; Warren et al., 2016; Lo et al., 2017; Sheahan et al., 2017; Brown et al., 2019; Sheahan et al., 2020). They include Coronaviridae (SARS-CoV, MERS-CoV, and bat coronaviruses), Paramyxoviridae (respiratory syncytial virus, Nipah virus, Hendra virus), and Filoviridae (Ebola virus) (Santoro and Carafoli, 2021; Warren et al., 2016; Lo et al., 2017; Sheahan et al., 2017; Brown et al., 2019; Sheahan et al., 2020). Coronavirus host specificity is determined by the variable spike glycoprotein, whereas the RdRp (nsp12) is highly conserved making it a suitable broad-spectrum target (Walls et al., 2020; Pruijssers et al., 2020). Sheahan et al. evaluated the drug’s *in vitro* antiviral activity in human airway epithelial (HAE) cultures infected with varied human and bat coronaviruses (Sheahan et al., 2017). In human CoV NL63, treatment produced a 3 log_10_ reduction in viral replication at 0.1 μM and complete inhibition at higher concentrations (Sheahan et al., 2017). Remdesivir also suppressed the replication of SARS-like and MERS-like bat CoVs, including the high-risk WIV1 and SHC014 strains (Sheahan et al., 2017).

Jorgensen et al. conducted early studies in 2020 to investigate remdesivir resistance in murine hepatitis virus (MHV), a coronavirus model with EC_50_ values similar to SARS-CoV-1, SARS-CoV-2, and MERS-CoV (Jorgensen et al., 2020). After repeated passaging under drug pressure, two RdRp mutations (F476L and V553L) were reported, conferring 2.4–5.6-fold reduced susceptibility *in vitro*. Nonetheless, the drug concentrations required to inhibit these variants remained below toxic levels, indicating that remdesivir would still be effective clinically (Jorgensen et al., 2020). These mutations carried a fitness cost, with wild-type virus rapidly outcompeting the variants, yet their conservation across coronaviruses suggested a potential shared resistance pathway (Jorgensen et al., 2020). By 2025, further studies focused directly on SARS-CoV-2, identifying multiple resistance-associated substitutions (RAS) within nsp12 (Fernandez-Antunez et al., 2025). Among these, S759A within the active site demonstrated more than a 100-fold increased replication under treatment, producing the strongest resistance observed (Fernandez-Antunez et al., 2025). Adjacent substitutions such as V166A/L, V792I, E796D, and C799F conferred intermediate effects (2–11.5-fold) while more distant mutations, including N198S, D484Y, and E802D, showed minimal or no resistance (≤2-fold) (Fernandez-Antunez et al., 2025). The data compiled from 2020 to 2025 has advanced knowledge of remdesivir resistance, revealing that while most resistance-associated substitutions confer only moderate decreases in susceptibility, active-site substitutions can mediate high-level resistance and warrant close surveillance (Jorgensen et al., 2020; Fernandez-Antunez et al., 2025).

### Pharmacokinetics

As a phosphoramidate prodrug, remdesivir undergoes extensive first-pass metabolism, thereby increasing the likelihood of poor oral bioavailability (Siegel et al., 2017). Remdesivir is exclusively administered by intravenous infusion, limiting its potential for prophylactic use (Chatterjee and Thakur, 2022). Due to these absorption limitations, Warren et al. evaluated the impact of route of administration on remdesivir’s efficacy in Ebola virus-infected rhesus monkeys (Warren et al., 2015). Results showed that intramuscular (IM) administration (at 3 mg/kg) initiated shortly after systemic viremia resulted in only partial protection, with 50% of animals surviving (Warren et al., 2015). In contrast, intravenous (IV) infusion (at 10 mg/kg) given on day 3 post-infection produced 100% survival and led to a significant reduction in plasma viral RNA levels (up to 5 log10 copies/mL decrease compared to placebo) along with effective elimination of clinical manifestations of Ebola virus disease (Warren et al., 2015). These findings highlight the necessity of IV administration for achieving therapeutic drug exposure (Siegel et al., 2017; Chatterjee and Thakur, 2022; Warren et al., 2015).

Many studies have further assessed the pharmacokinetics and tissue distribution of remdesivir and its metabolites (Hu et al., 2021; Li et al., 2021). Following an intravenous dose of 20 mg/kg, Hu et al. found that the parent drug is rapidly metabolized in the blood, becoming barely detectable after 0.5 h, while its metabolites (RMP, RTP, and RN) endure longer with higher blood levels (Hu et al., 2021). The results showed extensive distribution of the parent drug across all examined tissues, with the highest concentration observed in the liver (15,732 h∙nmol/kg) then the lung (3,116 h∙nmol/kg) (Hu et al., 2021). While the liver is well known for its first-pass metabolism, the lungs also act as a first-pass filter for drugs administered via the venous route (Upton and Doolette, 1999). Since the lung is the primary site of COVID-19 infection, Li et al. emphasize the importance of administering drugs parenterally (inhalation or intravenous infusion) to bypass the hepatic first-pass effect (Li et al., 2021). Using intravenous infusion experiments, Hu et al. observed that the RTP-to-RMP ratio in lung tissue exceeded that in the liver by more than 350-fold, demonstrating significantly enhanced phosphorylation and drug activation in the lungs (Hu et al., 2021).

The general dose recommendation of remdesivir for the treatment of COVID-19 is a loading dose of 200 mg diluted in normal saline (0.9%) or 5% dextrose, administered over 60 min on day 1, followed by a diluted dose of 100 mg IV for the subsequent 9 days (Singh et al., 2020). This regimen is supported by pharmacokinetic data demonstrating that plasma exposure of remdesivir and its metabolites increase in a dose-proportional manner across the 3–225 mg dose range (Humeniuk et al., 2020). Although the parent drug does not accumulate with once-daily dosing due of its short half-life, its metabolite GS-441524 exhibits modest accumulation (around 1.9-fold) with repeated dosing and achieves steady state by day 4 (Humeniuk et al., 2020). These characteristics justify clinical trials using a loading dose followed by once-daily maintenance dosing (Singh et al., 2020; Humeniuk et al., 2020).

Remdesivir is primarily metabolized in the liver, with carboxylesterase 1 accounting for approximately 80% of its metabolism, while cathepsin A and CYP3A each contribute about 10% (Agency, 2021; Gilead Sciences, 2020). Following administration of a single 150-mg [14C]-remdesivir (100 μCi) dose to healthy male participants, mean total recovery of the radioactive dose was greater than 92%, consisting of approximately 74% and 18% recovered in urine and feces, respectively (Humeniuk et al., 2021). However, most of the dose recovered in the urine was in the nucleoside form GS-441524 (48.6%), confirming that renal clearance was a major pathway for elimination of this metabolite, while unchanged remdesivir accounted for only 10.3% (Humeniuk et al., 2021). In short, remdesivir clearance is largely nonrenal, whereas GS-441524 and GS-704277 undergo modest renal clearance with minor active secretion, consistent with their low protein binding (Humeniuk et al., 2021). *In vitro* studies further show that remdesivir has minimal potential for clinically relevant drug–drug interactions (Humeniuk et al., 2021; Blair, 2023). Although remdesivir is a weak inhibitor of CYP3A4 and some transporters (OATP1B1, OATP1B3, MATE1), its rapid clearance and IV administration limit clinical impact (Humeniuk et al., 2021; Nies et al., 2021; Gilead Sciences, 2023). As seen, remdesivir is mainly metabolized via esterase pathways, with only minor CYP3A involvement (Humeniuk et al., 2021). These characteristics define a pharmacokinetic profile dominated by rapid distribution, esterase-driven metabolism, metabolite-driven elimination, and low interaction potential (Humeniuk et al., 2021; Blair, 2023; Agency, 2021; Gilead Sciences, 2020; Nies et al., 2021; Gilead Sciences, 2023).

Overall, remdesivir requires intravenous administration due to low oral bioavailability (Siegel et al., 2017). Following infusion, the parent compound is rapidly eliminated from plasma and converted into its active triphosphate metabolite (Hu et al., 2021). Remdesivir has a wide tissue distribution, with the liver receiving the highest proportion, but the lung accumulating relatively more active metabolite, hence the importance of administering drugs parenterally (Hu et al., 2021; Li et al., 2021; Upton and Doolette, 1999). The drug’s metabolism is predominately driven by carboxylesterase 1, with minor contributions from cathepsin A and CYP3A (Agency, 2021; Gilead Sciences, 2020). While the systemic clearance of the parent compound is nonrenal, its nucleoside metabolites GS-441524 and GS-704277 are mainly eliminated in urine (Humeniuk et al., 2021). Despite weak inhibition of CYP3A4, remdesivir does not demonstrate any clinically relevant drug-drug interactions (Humeniuk et al., 2021; Blair, 2023). These features justify intravenous dosing, the use of a loading dose followed by daily maintenance and the finding that metabolites, rather than remdesivir itself, determine antiviral exposure (Siegel et al., 2017; Chatterjee and Thakur, 2022; Warren et al., 2015; Hu et al., 2021; Li et al., 2021; Upton and Doolette, 1999; Singh et al., 2020; Humeniuk et al., 2020).

## Pros

### Reduced time-to-recovery

Among the main clinical trials studying remdesivir in 2020, the Adaptive COVID-19 Treatment Trial (ACTT-1), sponsored by the National Institute of Allergy and Infectious Diseases (NIAID), proved critical for the emergency approval for the use of remdesivir (Saint-Raymond et al., 2020). Between February-April 2020, the clinical study enrolled 1,062 adults hospitalized for COVID-19 and showing signs of lower respiratory tract infection (Beigel et al., 2020). It was a double-blinded and placebo-controlled trial taking place in the United States, Denmark, the United Kingdom, Greece, Germany, Korea, Mexico, Spain, Japan, and Singapore (Beigel et al., 2020). 541 patients intravenously received a loading dose of 200 mg followed by 100 mg of remdesivir daily for 9 days or until hospital discharge or death (Beigel et al., 2020). Supportive care was given to all patients based on each trial site’s standard of care protocol (Beigel et al., 2020). The primary outcome assessed was time to recovery, determined as the first day after 28 days of enrollment on which the patient qualified for category 1, 2 or 3 on the eight-category ordinal scale (Beigel et al., 2020). Extending from 1 (not hospitalized and no limitations of activities) to 8 (death), this clinical tool is used to measure COVID-19 severity depending on hospitalization status, oxygen requirements and other therapeutic needs (Beigel et al., 2020). Patients in the remdesivir group recovered faster (median 10 days) than those on placebo (median 15 days) and had 1.5 times higher odds of improvement on the ordinal scale at day 15. Results also showed a lower mortality rate with remdesivir by day 29 (11.4% vs. 15.2% in the placebo group) (Beigel et al., 2020).

The ACTT-1 trial’s very limited but robust data allowed major regulatory authorities to authorize the urgent use of remdesivir despite other studies yielding ambiguous results (Saint-Raymond et al., 2020). At the beginning of the pandemic, several studies have used these findings to evaluate the efficacy of remdesivir, including Davies et al., who conducted a benefit-risk assessment incorporating data from other trials (Davies et al., 2020). Davies et al. concluded that for the time being, remdesivir had a favorable benefit-risk profile in severe COVID-19 but further data on safety was urgently needed in ongoing and future trials (Davies et al., 2020).

### Reduced hospitalization and mortality in high-risk outpatients

Further studies supported the improved clinical outcomes observed in the ACTT-1 trial. Between September 2020-April 2021, Gottlieb et al. enrolled 562 non-hospitalized COVID-19 patients, with symptom onset within the previous 7 days, and having at least one risk factor for disease progression (Gottlieb et al., 2022). The trial took place at 64 sites in the United States, Spain, Denmark and the United Kingdom (Gottlieb et al., 2022). In this double-blinded, placebo-controlled RCT, remdesivir was administered at 200 mg on day 1, followed by 100 mg on days 2 and 3 to 279 patients (Gottlieb et al., 2022). Efficacy was primarily assessed by hospitalization due to COVID-19 or death from any cause within 28 days (Gottlieb et al., 2022). A secondary endpoint included COVID-19-related medical visits or all-cause mortality by day 28 (Gottlieb et al., 2022). Results suggested a hospitalization risk reduced by 87% in the remdesivir group comparing to the placebo group (Gottlieb et al., 2022). There was also an 81% lower risk of COVID-related visits or death with remdesivir highlighted by the following data (Gottlieb et al., 2022). 0.7% of patients in the remdesivir group faced a COVID-19-related hospitalization or death in contrast to 5.3% of patients in the placebo group (Gottlieb et al., 2022). Furthermore, 1.6% of patients in the remdesivir group and 8.3% of patients in the placebo group had a COVID-19-related medically attended visit by day 28 (Gottlieb et al., 2022).

### Effectiveness in specific disease stages and patient populations

Remdesivir was especially found to be potent in certain stages of infection or populations with varying degrees of COVID-19 severity. Between September 2021-June 2020, Jittamala et al. conducted a phase 2, multicenter, open label, controlled, adaptive, pharmacometric platform trial in Thailand and Brazil (Jittamala et al., 2023). The study targeted 131 low-risk adult patients with early symptomatic COVID-19 (reported symptoms for ≤4 days), oxygen saturation ≥96%, and unaffected in their daily activities (Jittamala et al., 2023). Patients were intravenously given a loading dose of 200 mg followed by 100 mg of remdesivir for 5 days. The trial’s primary outcome was the rate of viral clearance (Jittamala et al., 2023). The treatment effect was measured as the percentage multiplicative change in this rate compared to the control arm, indicating the extent to which remdesivir accelerates viral clearance (Jittamala et al., 2023). The treatment group demonstrated a 42% mean acceleration in viral clearance and a one-third shortening in median viral clearance half-life relative to the control group (Jittamala et al., 2023). Jittamala et al. employed a standardized pharmacometric assessment to support these results, confirming remdesivir’s antiviral efficacy consistent with other potent antivirals (Jittamala et al., 2023). A new feature in this paper is the use of remdesivir in vaccinated patients during the period of delta and omicron variants of COVID-19 (Jittamala et al., 2023). These findings were further supported by a retrospective study conducted by Dobrowolska et al. between 2021 and 2022, which provided real-world evidence for the observed outcomes during the period of delta and omicron variants (Dobrowolska et al., 2023). The previous trials we have mentioned were conducted prior to the widespread distribution of COVID-19 vaccines and so were more likely to result in hospitalization and severe outcomes (Beigel et al., 2020; Gottlieb et al., 2022; Jittamala et al., 2023). The secondary endpoint was all-cause hospitalization for clinical deterioration until day 28 (Jittamala et al., 2023). None of the patients in the treatment group developed severe COVID-19 in contrast to one patient in the control arm who was briefly hospitalized 1 day after discharge due to chest pain and lethargy (Jittamala et al., 2023). Finally, the trial found no evidence of difference in antiviral efficacy between different COVID-19 variants- Delta and early Omicron at the time of study (Jittamala et al., 2023). This data further supports that remdesivir specifically targets the highly conserved viral RdRp (Jorgensen et al., 2020).

The Solidarity trial is a global pragmatic study evaluating multiple COVID-19 therapeutics (Ali et al., 2022). The Canadian Treatments for COVID-19 (CATCO) was a substudy led by Ali et al. between August 2020 and April 2021 (Ali et al., 2022). This randomized control trial also reinforced remdesivir’s greater efficacy in patients with the lowest severity of disease (Ali et al., 2022). It was open-label and pragmatic, recruiting 1,267 patients in 52 hospitals across Canada (Ali et al., 2022). The treatment group was given remdesivir in addition to standard of care and compared to the control group receiving only standard of care (Ali et al., 2022). The dosage of intravenous remdesivir was as follows: 200 mg on day 1 then 100 mg daily for 9 days (Ali et al., 2022). The primary outcome of the study was in-hospital mortality (Ali et al., 2022). The results found it to be 18.7% in the remdesivir group versus 22.6% in the control group, indicating a relative risk of 0.83 (Ali et al., 2022). At 60 days, mortality was 24.8% in the remdesivir group and 28.2% in the control group (Ali et al., 2022). Although the mortality rate is quantitatively lower in the remdesivir group, the trial was considered underpowered to demonstrate statistical significance on their primary outcome (Ali et al., 2022). Further secondary outcomes comprised new need for mechanical ventilation (for those not ventilated at baseline), clinical severity of illness based on the WHO ordinal scale, oxygen-free and ventilation-free days, and special safety outcomes (new hepatic dysfunction and new need for renal replacement therapy) (Ali et al., 2022). For patients not requiring mechanical ventilation at baseline, the new need for it was significantly lower in the remdesivir group (8%) than in the control group (15%) (Ali et al., 2022). Patients treated with remdesivir had significantly more mean oxygen-free days (15.9 ± SD 10.5) and mean ventilator-free days (21.4 ± SD 11.3) at day 28 than the standard of care group (14.2 ± SD 11.1; 19.5 ± SD 12.3, respectively) (Ali et al., 2022).

In contrast to the above studies conducted in patients with low-severity COVID-19, the ACTT-1 by Beigel et al. suggested a potential signal of benefit in patients on ECMO or mechanical ventilation (baseline ordinal score of 7), though the results were inconclusive (Beigel et al., 2020; Jittamala et al., 2023; Ali et al., 2022). The rate ratio of recovery for these patients was 0.8, but the median recovery time could not be assessed (Beigel et al., 2020). Beigel et al. hypothesized that the short follow-up period and wide confidence intervals in this subgroup limited the ability to conclude, and while results indicated the greatest benefit in patients with less severe symptoms (baseline ordinal score of 5), they do not rule out a potential effect in patients on mechanical ventilation or ECMO (Beigel et al., 2020).

### Reduced progression to intermittent mandatory ventilation (IMV) or death in high-risk profiles

Following the original ACTT-1 trial, Paules et al. conducted a *post hoc* analysis to further explore these inconclusive results (Paules et al., 2022). Their goal was to assess remdesivir’s effect on progression to invasive mechanical ventilation or death and to develop a new risk profile (Paules et al., 2022). Remdesivir was reported to significantly reduce the risk of progression to invasive mechanical ventilation or death, with a hazard ratio of 0.67 (95% CI, 0.52–0.87; *p* = 0.0023) (Paules et al., 2022). A new risk stratification model was developed, predicting outcomes better than oxygen requirement alone (AUC 0.73 vs. 0.53) (Paules et al., 2022). The model uses baseline oxygen requirement plus hematologic markers: platelet count, absolute lymphocyte count (ALC), and absolute neutrophil count (ANC) (Paules et al., 2022). Results concluded that remdesivir had the greatest benefits in high-risk patients across all oxygen categories (low platelets, low ALC, high ANC, ± oxygen need) (Paules et al., 2022).

### Acceptable safety profile

While early clinical trials had a major focus on the efficacy of remdesivir, other studies concentrated on the adverse effects (Sise et al., 2024). At the time of emergency use authorization, remdesivir was not advised in patients with renal dysfunction (estimated glomerular rate (eGFR) less than 30 mL/min/1.73 m^2^) unless the therapeutic advantage outweighed the risks (Sise et al., 2024). REDPINE was the first phase 3 randomized, double-blind, placebo-controlled trial to assess remdesivir use in patients with severe kidney impairment (Sise et al., 2024). Between March 2021 and March 2022, the clinical study enrolled 243 hospitalized patients with COVID-19 pneumonia and either acute kidney injury, chronic kidney disease, or kidney failure (Sise et al., 2024). The clinical trial took place in 55 centers across 5 countries, including Brazil, Portugal, Spain, the United Kingdom, and the United States (Sise et al., 2024). Patients were randomized in a 2:1 ratio to intravenously receive remdesivir (a loading dose of 200 mg on day 1 followed by 100 mg for 4 days) or placebo (Sise et al., 2024). The findings showed that remdesivir was well tolerated, with no significant differences in adverse events between the remdesivir and placebo groups (Sise et al., 2024). Furthermore, kidney-related complications and changes in creatinine trends were similar among the groups (Sise et al., 2024). Moreover, the study showed no accumulation of remdesivir metabolites or its excipient (SBECD), allowing its use without dose adjustment in patients with eGFR <30 mL/min/1.73 m^2^ (Sise et al., 2024).

The safety of remdesivir use was further evaluated by van Laar et al. through a retrospective study of 103 hospitalized COVID-19 patients in the Netherlands (van Laar et al., 2021). Van Laar et al. analyzed the data of adult patients on a regular ward with oxygen supplementation who started a 5-day remdesivir treatment between August-November 2020 (van Laar et al., 2021). Around 20% of these patients had pre existing kidney and liver dysfunction and would have been excluded from randomized clinical trials (van Laar et al., 2021). The study compared renal and liver function at the start of treatment to values recorded during a 15-day follow-up period after remdesivir initiation (van Laar et al., 2021). Results suggested no cases of severe kidney damage, and only 11% of patients experienced a drop in eGFR greater than 10 mL/min/1.73 m^2^ (van Laar et al., 2021). Furthermore, the study showed mild liver abnormalities, notably 25% and 35% of patients experienced a drop in alanine transaminase and aspartate transaminase, respectively (van Laar et al., 2021). However, severe adverse events were rare (van Laar et al., 2021). These findings suggest that remdesivir can be used in patients with kidney and liver dysfunction, given that renal and hepatic functions are regularly monitored (van Laar et al., 2021).

Cheng et al. conducted a secondary analysis of the CATCO randomized trial by Ali et al. (2022) and Cheng et al. (2022). The primary outcome was to examine the risk of kidney or hepatic toxic effects with remdesivir administration, through changes in kidney function (eGFR) and hepatic function (ALT) at day 5 (Cheng et al., 2022). Among the 1281 adults enrolled in the CATCO, data was extracted for the 59 renally impaired patients (eGFR less than 30 mL/min/1.73 m^2^) (Ali et al., 2022; Cheng et al., 2022). The findings showed that patients treated with remdesivir had a higher median eGFR (29.2 vs. 16.5 mL/min/1.73 m^2^), however there was no evidence of worsened renal or hepatic injury (Cheng et al., 2022). Furthermore, median ALT levels were similar between groups, suggesting that remdesivir can be safely administered in patients with kidney dysfunction without dose modifications (Cheng et al., 2022).

The safety of remdesivir administration in renally impaired patients was initially challenged by studies carried out at the start of the pandemic, including a phase 3 open-label randomized controlled trial by Goldman et al. (2020). The trial recruited 397 hospitalized COVID-19 patients (≥12 years), with radiographic evidence of pulmonary infiltrates, and either an oxygen saturation ≤94% on ambient air or receiving supplemental oxygen, in the United States, Italy, Spain, Germany, Hong Kong, Singapore, South Korea, and Taiwan (Goldman et al., 2020). The first group intravenously received a loading dose of 200 mg followed by 100 mg of remdesivir for 4 days, while the second group kept taking 100 mg of remdesivir for 9 days after the initial loading dose (Goldman et al., 2020). Grade 4 decreases in creatinine clearance were observed in 12% of the 10-day group compared with 3% of the 5-day group, highlighting the increased risk of severe renal impairment with longer treatment (Goldman et al., 2020). However, the groups were not balanced at baseline in terms of disease severity, so the increased frequency in the 10-day group may be partly explained by their significantly worse clinical status since COVID-19 is known to cause renal injury (Goldman et al., 2020). Moreover, the major limitation of this study is the lack of a placebo control, unlike the more recent studies we’ve mentioned (Sise et al., 2024; van Laar et al., 2021; Cheng et al., 2022; Goldman et al., 2020).

Beyond renal and hepatic toxicity, additional studies evaluated safety in terms of general adverse effects (Gottlieb et al., 2022). Gottlieb et al. recruited patients with at least one preexisting risk factor (such as hypertension, cardiovascular disease, diabetes mellitus, obesity …) for progression to severe COVID-19 or aged 60 years and above (Gottlieb et al., 2022). The primary safety outcome assessed during the trial was determined by the emergence of adverse effects (Gottlieb et al., 2022). Results showed that 42.3% of patients in the remdesivir group and 46.3% of patients in the placebo group suffered from adverse events (Gottlieb et al., 2022). These findings confirmed the favorable safety profile of remdesivir in high-risk outpatients, supporting its use as a treatment for COVID-19 (Gottlieb et al., 2022).

Drug-induced QT prolongation is observed in a wide range of medications. Remdesivir can increase field potential duration with decreased Na+ peak amplitudes and spontaneous beating rates in a dose-dependent manner that might induce prolonged QT interval (Nabati and Parsaee, 2022). However, a few studies found no evidence of QT interval prolongation with remdesivir, including a historical cohort study by Saffar et al. (2024) and a basic science study by Szendrey et al. (2021).

### Ongoing trials in high-risk and immuncocompromised patients

Additional support for early remdesivir use in high-risk populations comes from an ongoing proof-of-concept interventional trial evaluating a 10-day course of remdesivir in asymptomatic or mildly symptomatic SARS-CoV-2–positive kidney transplant recipients (Gilead, 2025). This study hypothesizes that pre-emptive antiviral therapy initiated around the time of transplantation may prevent progression to severe COVID-19, allowing safer transplantation in immunosuppressed patients (Gilead, 2025). Although efficacy results are pending, the trial underscores growing interest in early and prolonged remdesivir administration in populations with altered immune responses and organ vulnerability, further extending its potential role beyond traditional hospitalized cohorts (Gilead, 2025).

Beyond monotherapy, emerging clinical trials are now evaluating combination antiviral strategies in high-risk populations (An Interventional Efficacy and Safety, 2025). A phase 3 randomized, double-blind trial is currently investigating ibuzatrelvir alone or in combination with remdesivir versus remdesivir alone in severely immunocompromised adults with early symptomatic COVID-19 (An Interventional Efficacy and Safety, 2025). Notably, remdesivir remains the active comparator and therapeutic backbone in this study, reflecting its continued clinical relevance despite heterogeneous efficacy signals across prior trials (An Interventional Efficacy and Safety, 2025). The focus on combination or extended antiviral regimens highlights recognition that select populations particularly immunocompromised patients—may require intensified treatment strategies rather than reliance on remdesivir monotherapy alone (An Interventional Efficacy and Safety, 2025).

The clinical trials discussed are comprehensively listed and summarized in Table 1.

**TABLE 1:** Clinical trials supporting the use of remdesivir in the treatment of COVID-19.

Study reference	Study design	Country	Intervention	Sample size	Dosage of remdesivir (intravenous)	Outcomes
Beigel et al. (2020)	RCT – double blinded, placebo-controlled	United States, Denmark, the United Kingdom, Greece, Germany, Korea, Mexico, Spain, Japan, and Singapore	Remdesivir treatment in hospitalized COVID-19 adult patients, with evidence of lower respiratory tract infection	1,062	200 mg, followed by 100 mg daily for up to 9 days.	Shorter recovery time and lower mortality with remdesivir. -Fewer support days needed by patients on ECMO or mechanical ventilation.
Gottlieb et al. (2022)	RCT – double-blinded, placebo-controlled	United States, Spain, Denmark, United Kingdom	Remdesivir treatment in non-hospitalized COVID-19 patients, with symptom onset within the previous 7 days, and having at least one risk factor for disease progression.	562	200 mg on day 1, followed by 100 mg on days 2 and 3	Hospitalization risk reduced by 87%, as well as an 81% lower risk of COVID-related visits or death with remdesivir. An acceptable safety profile is also recognized.
Ahmed et al. (2024)	Phase 2/3, open-label trial	Italy, Spain, United Kingdom, and United States	Remdesivir treatment in children infected with COVID-19.	53	Doses determined using physiologically based pharmacokinetic modeling: for ≥40 kg, 200 mg day 1, then 100 mg/day; for age ≥28 days and ≥3 to <40 kg, 5 mg/kg day 1, then 2.5 mg/kg/day	Clinical recovery observed in 62% of patients at day 10, followed by an 83% recovery rate at the last assessment. Pediatric drug exposure comparable to adults revealing no new safety concerns.
Jittamala et al. (2023)	RCT – phase 2 multicenter, open-label, controlled, adaptive, pharmacometric platform trial	Thailand and Brazil	Remdesivir treatment in low-risk adult patients with early symptomatic COVID-19	131	200 mg, followed by 100 mg daily for 5 days	Antivirals such as remdesivir are most efficient during early stages of infection with high viral load as opposed to later stages.
Ali et al. (2022)	RCT – open-label, pragmatic	Canada	Remdesivir plus standard care treatment versus standard care alone in randomized COVID-19 patients.	1,267	200 mg, followed by 100 mg daily for 9 days.	Remdesivir, in combination with standard care, is most beneficial to patients with lower severity of disease. Positive outcomes include lower risk of progression towards usage of mechanical ventilation as well as improved clinical status after 2 weeks.
Sise et al. (2024)	RCT – phase 3, double-blinded, placebo-controlled	Brazil, Portugal, Spain, the United Kingdom, and the United States	Remdesivir treatment in COVID-19 patients (aged 12 and above) hospitalized for pneumonia with acute kidney injury, chronic kidney injury, or kidney failure	243	200 mg, followed by 100 mg daily for up to 5 days.	No evidence of kidney-related adverse effects. Stable creatinine levels noted.
van Laar et al. (2021)	Retrospective study	Netherlands	Evaluation of liver and renal function in COVID-19 adult patients on regular ward with oxygen supplementation, and who began a 5-day remdesivir treatment.	103	-	Mild nephrotoxicity recorded with 11% of patients facing a decrease in eGFR. Mild hepatotoxicity also reported with 25% and 35% of patients exhibiting increase in alanine aminotransferase and aspartate aminotransferase levels, respectively. Findings suggest it is safe to reconsider and potentially lift absolute contraindications in patients with renal and/or hepatic impairment.
Cheng et al. (2022)	Secondary analysis of RCT – open-label, pragmatic	Canada	Remdesivir plus standard care treatment versus standard care alone in randomized COVID-19 patients.	59	200 mg, followed by 100 mg daily for 9 days.	No difference in mortality outcomes observed. No detected increase in risk of nephrotoxicity or transaminitis at day 5. The study supports safe administration of remdesivir in patients with renal impairment.

## Cons

### Mixed or neutral findings across major trials

As of the 1st of May 2020, the emergency use of remdesivir was approved by the FDA despite limited data (Saint-Raymond et al., 2020). The ACTT-1 had positive findings supporting this decision, while other studies had statistically insignificant results (Saint-Raymond et al., 2020; Beigel et al., 2020; Sise et al., 2024; Wang et al., 2020). Wang et al. carried out a randomized, double-blind, placebo-controlled, multicenter trial in China between February-March 2020 (Wang et al., 2020). They recruited 237 adult (≥18 years) hospitalized patients, RT-PCR positive for SARS-CoV-2, with pneumonia confirmed by chest imaging, and an oxygen saturation of 94% or lower on room air or a ratio of arterial oxygen partial pressure to fractional inspired oxygen of 300 mm Hg or less, and having symptoms for up to 12 days at most (Wang et al., 2020). The primary outcome was the time to clinical improvement within 28 days, measured as the number of days from randomization until either a two-level reduction on a six-point ordinal clinical status scale (1 = discharged to 6 = death) or discharge alive from hospital (Wang et al., 2020). The primary analysis was conducted in the intention-to-treat (ITT) population, while the safety analysis included all patients who began the assigned treatment (Wang et al., 2020). Findings indicated no statistically significant differences between the treatment and placebo groups in time to clinical improvement (Wang et al., 2020). It was 21 days in the remdesivir group versus 23 days in the placebo group (Wang et al., 2020). No statistically significant differences were apparent between the two groups in terms of duration of oxygen support, hospitalization length, interval from randomization to discharge, or interval from randomization to death (Wang et al., 2020). As for safety, adverse events occurred in 102 of 155 patients receiving remdesivir (66%) and in 50 of 78 patients receiving placebo (64%), indicating no substantial difference in the overall incidence of adverse events between the groups (Wang et al., 2020). Overall, no new safety concerns were identified (Wang et al., 2020). The trial was discontinued early due to the adverse effects in patients, thus not reaching the pre-determined sample size and reducing the statistical power of the study (Wang et al., 2020).

In the general population, some studies reported no statistically significant clinical differences between patients treated with remdesivir and those receiving standard care (Pan et al., 2021; Spinner et al., 2020). In an open-label randomized control trial, Spinner et al. enrolled 584 hospitalized adult COVID-19 patients with evidence of moderate pneumonia from hospitals in the United States, Europe, and Asia (Spinner et al., 2020). Between March and May 2020, the first group of patients was given 200 mg of remdesivir as a loading dose followed by 100 mg for 4 days, while the second group continued with 100 mg of remdesivir for 9 days after the loading dose (Spinner et al., 2020). The control group was treated according to the standard of care. The primary endpoint was the clinical status on day 11, assessed using a 7-point ordinal scale ranging from category 1 (death) to category 7 (discharged) (Spinner et al., 2020). No measurable clinical difference was observed between the 10-day remdesivir regimen and standard care (Spinner et al., 2020). As for the 5-day course, patients on remdesivir had improved clinical outcomes (odds ratio 1.65; 95% Cl, 1.09–2.48; P = 0.02) but these results failed to demonstrate statistical significance (Spinner et al., 2020). Analysis revealed no significant variations across the groups with respect to secondary outcomes, including duration of oxygen therapy or hospitalization, all-cause mortality at day 28 (1% in the 5-day remdesivir group, 2% in the 10-day group, and 2% in the standard care group), and time to recovery, among others (Spinner et al., 2020).

Between March and October 2020, Pan et al. undertook an international randomized open-control trial known as the WHO Solidarity Trial (Pan et al., 2021). 11,330 hospitalized COVID-19 patients were treated with standard of care alone or in combination with one of the following regimens: remdesivir, hydroxychloroquine, lopinavir alone, interferon alone, interferon with lopinavir (Pan et al., 2021). The dosage for remdesivir was a loading dose of 200 mg followed by 100 mg daily for 9 days. The primary goal was to assess in-hospital mortality (death during the index hospitalization; follow-up ended at discharge), irrespective of whether death happened before or after day 28 (Pan et al., 2021). Secondary outcomes were limited to initiation of mechanical ventilation and hospitalization duration (Pan et al., 2021). The study found equivalent results for all outcomes between the patients taking remdesivir and the control group. In terms of in-hospital mortality, 12.5% of the patients on remdesivir died versus 12.7% in the control group, indicating a rate ratio of 0.95 (95% confidence interval [CI], 0.81 to 1.11; P = 0.50) (Pan et al., 2021).

Paules et al. found that remdesivir is beneficial in patients requiring supplemental oxygen, but Ader et al. demonstrated otherwise (Paules et al., 2022; Ader et al., 2022). Between March 2020 and January 2021, they conducted a phase 3 randomized controlled open-label study to evaluate the clinical efficacy of remdesivir in hospitalized patients with COVID-19 (Ader et al., 2022). This DisCoVeRy trial included 857 patients from 48 different hospitals in France, Belgium, Austria, Portugal, and Luxembourg (Ader et al., 2022). Moreover, this trial focused on COVID-19 patients suffering from hypoxemic pneumonia or requiring oxygen supplementation (Ader et al., 2022). Patients were randomized to receive standard care alone or in combination with remdesivir as follows: 200 mg on day 1, then 100 mg daily for 9 days (Ader et al., 2022). The primary outcome was the clinical status of patients at day 15, measured by the WHO seven-point ordinal scale, starting with 1 for nonhospitalized until 7 for death (Ader et al., 2022). Findings of this study showed no significant differences in the clinical status of patients receiving remdesivir compared to the control group (Ader et al., 2022). In fact, in the remdesivir group: 15% were discharged without limitations (vs. 17% in control group); 31% were discharged with limitation (vs. 32% on control group); 12% were hospitalized without oxygen demand (vs. 7% in control group); 18% were hospitalized and required oxygen supplementation (vs. 16% in control group); 15% needed invasive mechanical ventilation (vs. 19% in control group) (Ader et al., 2022). Furthermore, the duration of hospitalization and mortality were similar between the two groups (Ader et al., 2022). Therefore, this trial undermines the efficacy of remdesivir to improve clinical outcomes of COVID-19 patients suffering from hypoxemic pneumonia or requiring oxygen supplementation (Ader et al., 2022).

While the REDPINE trial confirmed the safe use of remdesivir in COVID-19 patients suffering from renal impairment, it, on the other hand, failed to show its efficacy in the treatment of the latter population (Sise et al., 2024). As mentioned previously, the REDPINE trial, a phase 3 randomized, double-blind, placebo-controlled trial, analyzed the use of remdesivir in patients with severe kidney impairment. Patients were randomized in a 2:1 ratio to receive remdesivir or placebo (Sise et al., 2024). The primary outcome was assessed by all-cause mortality and the need for invasive ventilation (Sise et al., 2024). In fact, composite all-cause mortality or the need for invasive ventilation by day 29 were 29.4% and 32.5% in the remdesivir and placebo groups respectively (Sise et al., 2024). These findings undermine the efficacy of remdesivir as a treatment for COVID-19 in patients with renal impairment (Sise et al., 2024).

### Single-center study suggesting longer recovery

Contrary to the studies in the pros section of this paper, Sellers et al. found remdesivir to increase recovery time and hospital stay in some patients (Sellers et al., 2023). They studied the clinical efficacy of remdesivir in the treatment of moderately to severely hospitalized patients with COVID-19 (Sellers et al., 2023). The study was a single-center retrospective cohort analysis. This study included 300 patients of which 200 received remdesivir and standard of care, while the others received standard of care alone (Sellers et al., 2023). Remdesivir was administered with a starting loading dose followed by daily infusions (Sellers et al., 2023). The primary outcome was time needed to recovery assessed through hospital discharge or discontinued oxygen supplement (Sellers et al., 2023). Moreover, Sellers et al. used the 4C mortality score categories to stratify patients’ outcomes as low, intermediate, high, and very high risk (Sellers et al., 2023). Findings showed that in the intermediate and high-risk group recovery time was longer in the remdesivir group compared to standard of care alone (Sellers et al., 2023). Moreover, no significant difference was found in the low and very high-risk group (Sellers et al., 2023). Furthermore, mortality rate did not differ between the 2 groups (Sellers et al., 2023).

### Possible adverse effects

**FIGURE 3:**
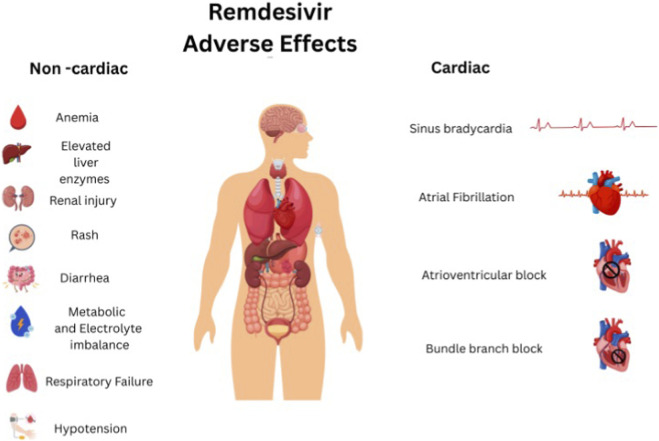
Remdesivir adverse effects (created on Canva).

Remdesivir has been associated with a wide range of adverse effects that can be divided into two categories: non-cardiac and cardiac (Nabati and Parsaee, 2022). Non-cardiac adverse events include anemia, elevated liver enzymes, renal injury, rash, diarrhea, metabolic and electrolyte imbalances, hypotension, respiratory failure, and multi-organ dysfunction (Gupta et al., 2020). Importantly, several cardiac effects have been reported ranging from sinus bradycardia, atrial fibrillation to conduction abnormalities like atrioventricular block and bundle branch block (Nabati and Parsaee, 2022). A summary of the adverse effects is provided in Figure 3.

The clinical trials discussed are comprehensively listed and summarized in Table 2.

**TABLE 2:** Clinical trials showing limited or no benefit of remdesivir in the treatment of COVID-19.

Study	Study design	Country	Intervention	Sample size	Dosage of remdesivir (intravenous)	Outcomes
Wang et al. (2020)	RCT – double-blinded, placebo-controlled, multicentre	China	Remdesivir treatment in adults (≥18 years) hospitalized with confirmed COVID-19, ≤12 days from symptom onset, SpO_2_ ≤94% on room air or PaO_2_/FiO_2_ ≤300 mmHg and radiologically confirmed pneumonia.	237	200 mg on day 1, followed by 100 mg daily for up to 9 days.	Quicker clinical improvement observed in patients taking remdesivir, but the results are not statistically significant.
Goldman et al. (2020)	RCT – phase 3, open-label	United States, Italy, Spain, Germany, Hong Kong, Singapore, South Korea, and Taiwan.	Remdesivir treatment in hospitalized COVID-19 patients (≥12 years), with radiographic evidence of pulmonary infiltrates, and either an oxygen saturation ≤94% on ambient air or receiving supplemental oxygen.	397	Group 1: 200 mg on day 1, followed by 100 mg daily for 4 days Group 2: 200 mg on day 1, followed by 100 mg daily for 9 days	Comparing 5–10 days of remdesivir so not very relevant for us. We want to compare remdesivir to no remdesivir. So probably will remove this. Using this source in the pros section for the renal part.
Spinner et al. (2020)	RCT – open-label	United States, Europe, and Asia.	Remdesivir treatment in hospitalized adult COVID-19 patients with evidence of moderate pneumonia	584	200 mg on day 1, followed by 100 mg daily for 4 days in group 1 and 9 days in group 2	No measurable clinical difference observed between patients taking 10 days of remdesivir and those treated with standard of care. Improved clinical outcomes seen with the 5-day remdesivir regimen but lack of clear clinical significance.
Ader et al. (2022)	RCT – phase 3, open-label, adaptive, multicenter	Europe	Remdesivir treatment in hospitalized COVID-19 adult patients with evidence of hypoxemic pneumonia or requiring oxygen supplementation	857	200 mg on day 1, followed by 100 mg daily for up to 9 days.	No clinical improvement documented in COVID-19 hospitalized patients, having symptoms for more than a week, and on oxygen support when taking remdesivir plus standard of care versus standard of care alone.
Pan et al. (2021)	RCT – open control	International	Treatment of hospitalized COVID-19 patients with standard care or one of the following regimens: remdesivir, hydroxychloroquine, lopinavir alone, interferon alone, interferon with lopinavir.	11,330	200 mg on day 1, followed by 100 mg daily for 9 days.	Equivalent outcomes reported in hospitalized COVID-19 patients taking remdesivir versus the control group in terms of mortality, duration of hospital stay, and initiation of ventilation.
Sellers et al. (2023)	Retrospective cohort study – single-center	-	Evaluation of time to recovery in hospitalized patients with moderate-to-severe COVID-19	300	-	An increase in recovery time and hospital stay observed in COVID-19 patients of the intermediate and high risk mortality groups, receiving remdesivir. No significant differences between the remdesivir and standard of care groups for patients in the low and very high risk mortality divisions.
Sise et al. (2024)	RCT – phase 3, double-blinded, placebo-controlled	Brazil, Portugal, Spain, the United Kingdom, and the United States	Remdesivir treatment in COVID-19 patients (aged 12 and above) hospitalized for pneumonia with acute kidney injury, chronic kidney injury, or kidney failure	243	200 mg on day 1, followed by 100 mg daily for up to 5 days.	No significant difference in mortality outcomes and usage of mechanical ventilation in patients with renal impairment treated with remdesivir versus standard of care.

**FIGURE 4:**
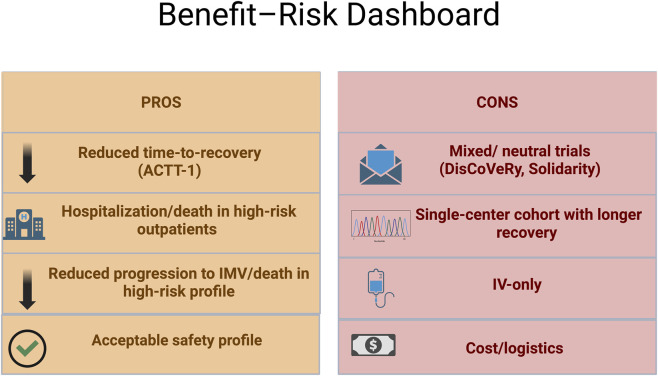
Benefit-risk dashboard of remdesivir *created by biorender.com. This figure provides a comparative summary of remdesivir’s clinical benefits and limitations across key randomized and real-world studies. On the benefit side, randomized controlled trials demonstrate reduced time to recovery (ACTT-1) and lower risk of hospitalization, progression to invasive mechanical ventilation, or death in high-risk patients, particularly when administered early and before advanced respiratory failure. These benefits appear stage-dependent, with diminished effects in critically ill or mechanically ventilated populations. On the limitation side, large pragmatic trials (e.g., DisCoVeRy, WHO Solidarity) reported neutral clinical outcomes, highlighting heterogeneity driven by disease severity, trial design, and timing of therapy. Observational cohorts further suggest variable recovery trajectories in select subgroups. Practical constraints, including intravenous-only administration, cost, and logistical barriers, limit broad outpatient applicability despite demonstrated antiviral activity.

Figure 4 highlights a benefit-risk profile for the use of remdesivir. summarizing the pros and cons discussion above.

## Reconciling the evidence

Conflicting findings emerged across the clinical studies appraised in this paper due to differences in the following parameters: timing of remdesivir initiation, baseline disease severity, endpoint definitions, and statistical power. The trials in the pros section yielded more favorable results because they initiated treatment earlier than the trials in the cons section. For instance, Jittamala et al. began remdesivir treatment at a mean of 2.4 days after symptoms began, and Gottlieb et al. at a median of 5 days (Gottlieb et al., 2022; Jittamala et al., 2023). Conversely, in the studies carried out by Spinner et al. and Wang et al., discussed in the cons section, treatment started after a median of 8–9 days and 10–11 days respectively (Wang et al., 2020; Spinner et al., 2020). Furthermore, the studies of the pros section targeted patients who were less critically ill, thus using remdesevir to prevent deterioration rather than curing severe illness. Indeed, the ACTT-1 trial found the most evident benefit in patients receiving low flow-oxygen (ordinal score of 5) (Beigel et al., 2020). However, since Wang et al. included a large proportion of patients with hypoxemic pneumonia or advanced respiratory failure, the positive findings in the less severely ill patients were diluted (Wang et al., 2020). It is also important to recognize that studies had defined different endpoints. While the pros section had endpoints focused on early treatment-responsive changes such as time to recovery in the ACTT-1 study, the cons section prioritized survival and clinical improvement (Beigel et al., 2020). Studies in the pros section were more likely to demonstrate positive findings because their selected outcomes occur early in the disease course, before progression to severe illness, allowing antiviral therapy a greater opportunity to show benefit. Finally, the studies that reported negative or insignificant results were largely constrained by limitations in statistical power. For example, the trial carried out by Wang et al. was severely underpowered due to its power declining from 80% to 58%, forcing it to end early (Wang et al., 2020).

## A focus on patients with congenital heart disease (CHD)

Most clinical trials testing the efficacy of remdesivir were conducted in adults. Limited pediatric data suggest potential benefits, but the mechanisms and optimal use in patients with underlying cardiovascular conditions remain underexplored.

Ahmed et al. ran a phase 2/3 open-label trial between July 2020 and May 2021 in Italy, Spain, the United States, and the United Kingdom (Ahmed et al., 2024). The study recruited 53 pediatric patients aged 28 days to 17 years old and hospitalized for COVID-19 (Ahmed et al., 2024). Notably, 21% had cardiac disorders, highlighting the importance of evaluating antiviral therapy in this subgroup (Ahmed et al., 2024). Doses were determined using physiologically based pharmacokinetic modeling: for ≥40 kg, 200 mg day 1, then 100 mg/day; for age ≥28 days and ≥3 to <40 kg, 5 mg/kg day 1, then 2.5 mg/kg/day (Ahmed et al., 2024). Clinical recovery was reported in 62% of patients at day 10, followed by an 83% recovery rate at the last assessment (Ahmed et al., 2024). Moreover, remdesivir dosing achieved drug exposures comparable to adults, with no new safety concerns identified (Ahmed et al., 2024). Data from this study provided the evidence supporting the approval of remdesivir use in pediatric patients aged ≥28 days and weighing ≥3 kg, an indication that was added on 25 April 2022 (Ahmed et al., 2024; Chan-Tack et al., 2023).

Other studies further evaluated remdesivir’s safety in pediatric COVID-19 patients (Nieves et al., 2025; Khalil et al., 2023). In a retrospective cohort study enrolling 318 patients aged 5–18 years old, Nieves et al. concluded that remdesivir was safe and well tolerated in pediatric patients (Nieves et al., 2025). They found no clinically significant hematological or renal toxicity, and while liver enzymes increased modestly, they returned to baseline with continued treatment (Nieves et al., 2025). In another retrospective case-controlled study with 60 pediatric patients, 38 patients tolerated remdesivir well despite a few common adverse effects: hypoalbuminemia in 19 cases (50.0%) and anemia in 18 cases (47.4%) (Khalil et al., 2023). Khalil et al. inferred that remdesivir may be a safe and therapeutic option for children affected by COVID-19 (Khalil et al., 2023).

Data documenting the clinical impact of COVID-19 in patients witch CHD remain scarce, though some papers provide valuable insight and highlight research gaps (Sendzikaite et al., 2021; Lewis et al., 2020). Sendzikaite et al. published a review addressing frequently asked questions in pediatric and congenital cardiology (Sendzikaite et al., 2021). The authors noted that, despite limited evidence, children with CHD appear to have a considerably better prognosis than adults with CHD, though this may be partially explained by differences in cardiovascular reserve, immune response, and lower prevalence of comorbidities in pediatric populations (Sendzikaite et al., 2021). This observation was supported by a multicenter cross-sectional study evaluating ICU mortality in children with COVID-19, although only 6% of enrolled patients had CHD and most had other comorbidities (Shekerdemian et al., 2020). They reported an overall ICU mortality of less than 5% in children with COVID-19, compared with published ICU mortalities of 50%–62% in adults (Shekerdemian et al., 2020). Further data from China also support this finding (Dong et al., 2020; Lu et al., 2020).

Emerging evidence suggests that COVID-19 severity in patients with CHD is driven predominantly by underlying cardiovascular physiology rather than anatomical diagnosis alone (Broberg et al., 2021). CHD phenotypes associated with reduced cardiopulmonary reserve such as single ventricle circulation, cyanosis, pulmonary hypertension, and ventricular dysfunction appear to be at a higher risk of severe disease, intensive care admission, and cardiovascular decompensation (Haiduc et al., 2021). In these patients, chronic hypoxemia and fixed hemodynamic constraints may amplify COVID-19 symptoms (Haiduc et al., 2021).

These findings raise the question of whether the pathophysiology of CHD may modify remdesivir’s pharmacologic behavior and therapeutic effectiveness in COVID-19. Although remdesivir’s pharmacological properties have not been examined in hemodynamically unstable patients, studies such as that by Morales Castro et al., have shown that critical illness generally affects drug pharmacokinetics and pharmacodynamics (Morales Castro et al., 2023). Therefore conditions including reduced cardiac output, intracardiac shunting, and chronic hypoxemia seen in certain CHD phenotypes could limit antiviral efficacy of remdesivir. Furthermore, as described by Chung et al., the most severe cardiovascular complications—such as endothelial and microvascular damage and myocardial stress—often manifest during the late stages of the disease around day 10 (Chung et al., 2021). Thus, in CHD patients whose cardiopulmonary reserve is already limited, clinical deterioration occurs after peak viral replication, potentially narrowing the therapeutic window during which remdesivir provides maximal benefit. Finally, the marked variability in COVID-19 severity observed among patients with CHD, as reported by Haiduc et al., may significantly influence antiviral treatment response (Haiduc et al., 2021). Collectively, these considerations underscore the need for dedicated pharmacokinetic and clinical studies to determine how CHD-specific physiology may alter remdesivir’s therapeutic profile in COVID-19.

Although children with COVID-19 usually experience a mild disease course, those with CHD are at increased risk of severe manifestations, including worsened hypoxemia and impaired tissue perfusion (Zareef et al., 2020). The risk is further heightened in CHD patients with additional comorbidities such as depressed myocardial contractility, pulmonary hypertension, or immunodeficiencies (Zareef et al., 2020). In the absence of robust data, current guidelines recommend supportive and symptomatic treatment for COVID-19 in this population (Zareef et al., 2020). For instance, a 9-week-old patient with unrepaired balanced complete atrioventricular canal defect failed to respond to remdesivir, emphasizing the need for individualized treatment strategies and mechanistic studies exploring why antiviral therapy may be less effective in certain CHD phenotypes (Rodriguez et al., 2020).

Data on the outcomes associated with remdesivir use for the treatment of COVID-19 in adult congenital heart disease (ACHD) remain limited (Sendzikaite et al., 2021; ESC guidance for the diagnosis, 2022). To develop efficient treatment guidelines, ACHD patients are categorized into low-risk, intermediate-risk, and high-risk groups (Radke et al., 2020). Patients with complex cyanotic defects, palliated univentricular hearts, advanced heart failure, severe valvular disease, or pulmonary hypertension are categorized as high risk (Radke et al., 2020). During the pandemic, Radke et al. suggested that ACHD patients with low to moderate risk who showed no signs of deterioration could be monitored remotely and managed at home (Radke et al., 2020). In contrast, high-risk patients or those presenting with respiratory or cardiovascular compromise were advised to be admitted, preferably to a specialized tertiary ACHD center (Radke et al., 2020). Currently, the Adult Congenital Heart Association (ACHA) Medical Advisory Board recommends full COVID-19 vaccination for all adults with congenital heart disease, as well as for their family members and caregivers (ACHA, 2023).

## Conclusion

The rapid emergence of the COVID-19 pandemic emphasized the need to establish treatment protocols and develop a vaccine. Numerous clinical trials were conducted to evaluate remdesivir’s antiviral efficacy and adverse effects. Treatment with remdesivir was shown to shorten recovery time and reduce mortality risk in several randomized controlled trials (Beigel et al., 2020; Gottlieb et al., 2022). The drug appeared to be especially effective in patients at an early stage of infection or with mild disease severity (Jittamala et al., 2023; Ali et al., 2022). Additional findings suggested potential benefit in patients requiring ECMO, mechanical ventilation, or any form of supplemental oxygen (Beigel et al., 2020; Paules et al., 2022).

Early guidelines contraindicated the use of remdesivir in patients with severe renal impairment (eGFR <30 mL/min/1.73 m^2^) due to concerns about the excipient and the lack of safety data (U.S. Food and Drug Administration, 2020b; Agency, 2020). However, clinical studies such as the REDPINE trial (Sise et al., 2024) demonstrated its safety in this population, leading to an FDA label update that now allows remdesivir to be used without dose adjustment in patients with any degree of renal impairment (U.S. Food and Drug Administration, 2023). Some trials failed to demonstrate significant benefits (Pan et al., 2021; Spinner et al., 2020), with one even reporting an increase in recovery time and hospital stay (Sellers et al., 2023). However, these negative findings are outweighed by evidence from other trials showing improved clinical outcomes.

In patients with CHD, the effects of remdesivir, and even the impact of COVID-19 itself, remain uncertain highlighting an important research gap. Besides formulating new therapeutic agents, it is crucial to further develop existing drugs such as remdesivir which has already demonstrated considerable potential. In the meantime, physicians can refer to treatment guidelines established by the National Institutes of Health which provide guidance across different patient populations (National Institutes of Health, 2021).
